# Transient Receptor Potential Melastatin-4 Is Involved in Hypoxia-Reoxygenation Injury in the Cardiomyocytes

**DOI:** 10.1371/journal.pone.0121703

**Published:** 2015-04-02

**Authors:** Hulin Piao, Ken Takahashi, Yohei Yamaguchi, Chen Wang, Kexiang Liu, Keiji Naruse

**Affiliations:** 1 Department of Cardiovascular Surgery, the Second Affiliated Hospital of Jilin University, Changchun, China; 2 Department of Cardiovascular Physiology, Graduate School of Medicine, Dentistry and Pharmaceutical Sciences, Okayama University, Okayama, Japan; 3 Department of Cardiology, Graduate School, Dalian Medical University, Dalian, China; Virginia Commonwealth University, UNITED STATES

## Abstract

Ischemic heart disease still remains the most common cause of cardiac death. During ischemia-reperfusion (I/R), reactive oxygen species (ROS) are produced in excess in cardiac tissue, where they induce cell death. Our previous study showed that 9-phenanthrol (9-Phe), a specific inhibitor of the TRPM4 channel, preserves cardiac contractile function and protects the heart from I/R injury-related infarction in the excised rat heart. Accordingly, we hypothesized that TRPM4 channels are involved in the 9-Phe-mediated cardioprotection against ROS-induced injury. In rats, intravenous 9-Phe mitigated the development of myocardial infarction caused by the occlusion of the left anterior descending artery. Immunohistochemical analysis indicated that TRPM4 proteins are expressed in ventricular myocytes susceptible to I/R injury. Hydrogen peroxide (H_2_O_2_) is among the main ROS overproduced during I/R. In the cardiomyocyte cell line H9c2, pretreatment with 9-Phe prevented cell death induced by conditions mimicking I/R, namely 200 μM H_2_O_2_ and hypoxia-reoxygenation. Gene silencing of TRPM4 preserved the viability of H9c2 cardiomyocytes exposed to 200 μM H_2_O_2_. These results suggest that the cardioprotective effects of 9-Phe are mediated through the inhibition of the TRPM4 channels.

## Introduction

Ischemic heart disease is the most common type of heart disease causing cardiac death. Early and successful restoration of blood flow to an ischemic myocardium is the most effective strategy to improve clinical outcome. Treatments include thrombolytic therapy, percutaneous coronary intervention (PCI), and coronary artery bypass graft (CABG). However, the process of restoring blood flow to the ischemic area causes additional cell death by ischemia-reperfusion (I/R) injury. Therefore, I/R injury reduces the beneficial effects of myocardial reperfusion. Myocardial I/R cause many complications, such as arrhythmia, contractile dysfunction, and myocardial infarction [1]. Therefore, novel therapeutic strategies are required to protect the myocardium against I/R injury in patients with ischemic heart disease. Despite significant advances in our understanding of the mechanisms underlying this process, the current treatments for I/R injury remain rudimentary.

It is widely recognized that reactive oxygen species (ROS) play important roles in I/R injury [2–5]. During I/R, endothelial cells, leukocytes, and cardiomyocytes produce ROS as by-products of various signaling pathways (i.e., mitochondrial respiration) and enzyme activities such as xanthine oxidase, cytochrome oxidase, and cyclooxygenase [6]. ROS cause protein denaturation, the inactivation of key homeostatic enzymes, and peroxidation of lipid membranes. These highly detrimental processes cause the death of cardiomyocytes and myocardial infarction. Hydrogen peroxide (H_2_O_2_) is among the main ROS whose production is significantly increased during I/R [7].

Our previous study showed that a hydroxyl tricyclic derivative, 9-phenanthrol (9-Phe), exhibits cardioprotective properties against I/R, evidenced by reduced infarct size (IS) and preserved contractile function in isolated rat hearts [8]. We demonstrated that the cardioprotective effects of 9-Phe are not derived from the well-known mechanism of mitochondrial K_ATP_ channel opening. Therefore, the mechanism remains unknown. 9-Phe is the most specific inhibitor of the transient receptor potential melastatin-4 (TRPM4) channel [9, 10]. This compound has no effect on TRPC3 and TRPC6, as well as the Ca^2+^-activated K^+^, voltage-dependent K^+^, inward rectifying K^+^, and voltage-dependent Ca^2+^ channels. Therefore, we hypothesized that TRPM4 channels are involved in the 9-Phe-mediated cardioprotection against I/R injury.

In this study, we examined the cardioprotective effect of 9-Phe against I/R injury produced by occlusion of the left anterior descending artery (LAD) *in vivo*. Then, we investigated the expression of TRPM4 proteins in cardiac tissues by immunohistochemistry. To elucidate the cardioprotective mechanism of 9-Phe, we examined the impact of 9-Phe on H_2_O_2_ exposures using the rat cardiomyocyte cell line H9c2. Hypoxia-reoxygenation (H/R) challenge to the H9c2 cells was also used to mimic the cellular environment of I/R injury. Finally, we performed knockdown experiments of TRPM4 channels to clarify the involvement of these channels in the ROS-induced death of cardiomyocytes.

## Materials and Methods

### Animals

Adult male Sprague–Dawley rats (12–14 weeks old) were used in this study. All animal handling and experimental procedures were approved by the Animal Care and Use Committee of Okayama University; Permit: OKU-2013054). Animal surgery was performed under sodium pentobarbital anesthesia, and every effort was made to minimize suffering.

### In vivo animal experiment

**Fig 1:**
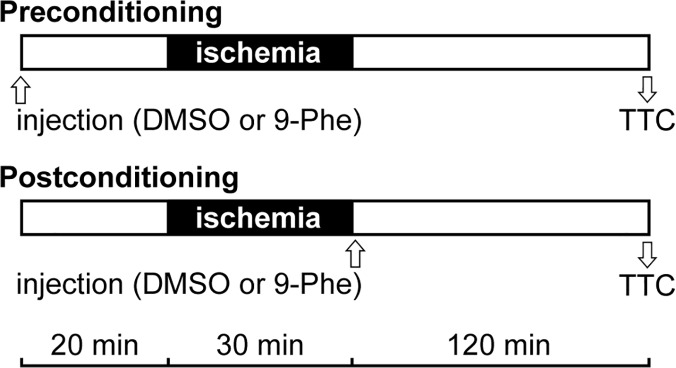
Animal experimental protocol. Rats were treated with the vehicle (DMSO; Groups 1 and 3) or 9-Phe (1 mg/kg in DMSO; Groups 2 and 4). The bolus injections into the jugular vein were given before (preconditioning; Groups 1 and 2) or after (postconditioning; Groups 3 and 4) ischemia. The 30-min ischemia period was followed by 120 min of reperfusion, and then the hearts were collected for TTC staining.

The rats were randomly divided into four groups. Group 1 and Group 3 were administered dimethyl sulfoxide (DMSO), whereas Group 2 and Group 4 were administered 9-Phe (1 mg/kg; dissolved in DMSO). All treatments were single intravenous bolus injections into the jugular vein. Whereas Groups 1 and 2 were treated 20 min before ischemia (preconditioning), Groups 3 and 4 were treated at the onset of reperfusion (postconditioning; Fig 1).

Anesthesia was induced by an intraperitoneal injection of pentobarbital (50 mg/kg). Surgery was performed under artificial ventilation using a small animal ventilator SAR1000 (CWE, USA), and the body was maintained at normal temperature. Left thoracotomy was performed at the fourth intercostal space, and LAD was occluded with a 5–0 prolene suture. The rats were subjected to 30 min ischemia by ligating the artery, followed by 2 h of reperfusion.

### Evans blue staining and TTC staining

After the 2-h reperfusion period, the heart was rapidly removed for histopathology study. LAD was re-occluded, and the heart was infused with 2 ml of 5% Evans blue via the aorta to distinguish the ischemic and non-ischemic areas. The heart was rinsed with PBS, frozen at −80°C for 60 min, and cut into 2-mm thick transverse sections. Each slice was incubated in 1% 2,3,5-triphenyltetrazolium chloride (TTC) dye (10 min; 37°C), followed by 10-min neutralization in 4% paraformaldehyde at room temperature. Digital photographs of the transverse sections were taken. The infarct size, area at risk (AAR), and the percentage of the infarct size to AAR were determined using image J software [11].

### Immunohistochemistry and immunofluorescence assay

The heart, kidney, liver, lung, and pancreas of a 13-week-old male rat were collected under pentobarbital anesthesia. The specimens were fixed in 4% paraformaldehyde, and embedded in paraffin. Serial 4-μm-thick sections were cut from paraffin-embedded tissue blocks. For immunohistochemistry, sections were stained using an automated Bond Max stainer (Leica Biosystems, Melbourne, VIC, Australia). Anti-TRPM4 antibody (1:400; ACC-044; Alomone Labs, Jerusalem, Israel) was used as the primary antibody, and the TRPM4-positive cells were detected by 3,3′-Diaminobenzidine (DAB) staining. Negative control sections were treated similarly in the absence of primary antibodies, and then stained with DAB.

For immunofluorescence assays, paraffin sections were stained with anti-TRPM4 (1:250; ab63080, Abcam, Tokyo, Japan) overnight at 4°C. Then, the sections were washed and incubated with Alexa Fluor 488-conjugated secondary antibody (1:10000; ab150077, Abcam) for 1 h at room temperature, washed once with PBS, and incubated with Hoechst 33342 (1:1000; Abcam) for 1 min to stain the nuclei. To test the specificity of the anti-TRPM4 antibody, the primary antibody was preincubated 30 min with the antigenic blocking peptide (ab65597; Abcam) prior to use. Fluorescence images were obtained using a confocal microscope (LSM 780, Zeiss, Germany). They were all blinded to the experimenter and acquired with identical parameters.

For double immunofluorescence assays, paraffin sections were stained with the primary antibodies anti-TRPM4 (1:250; ab63080, Abcam) and SERCA2 (1:200; ab2861, Abcam). A mixture of two secondary antibodies (Alexa Fluor 488-conjugated anti-rabbit IgG and Alexa Fluor 568-conjugated anti-mouse IgG) was used at dilution of 1:10000.

### Cell culture

The H9c2 cell line was purchased from American Type Cell Culture (ATCC, Manassas, VA, USA). Cells were cultured in DMEM (Invitrogen, Carlsbad, CA, USA) supplemented with 10% fetal bovine serum (FBS) in a humidified incubator (5% CO_2_; 37°C), and used at 80%–90% confluence.

### Analysis of 9-Phe protective effects against oxidative stress by MTT assay

Viability of the H9c2 cells was quantified using the MTT Cell Proliferation Assay Kit (Cayman Chemical Company, Ann Arbor, MI, USA). Before the assay, cells were trypsinized and seeded into a 96-well culture plate (10,000 cells/well) for 24 h to reach 80% confluence. Then, wells were incubated with DMSO (control) or 9-Phe (1, 2, 5, 10, or 20 μM), according to the following protocols. The first set of experiments tested the protective effect of 9-Phe against H_2_O_2_-induced cell injury. The cultures were incubated in the presence of 200 μM of H_2_O_2_ for 4 h after the addition of DMSO or 9-Phe. The second set of experiments used the H/R model, whereby culture medium was replaced with serum-free medium, and the cells were incubated under hypoxic conditions (95% N_2_; 5% CO_2_) for 4 h at 37°C. After the hypoxia treatment, the cells were cultured in DMEM supplemented with 10% FBS under normoxic conditions for 1 h. After both sets of experiments, each well was incubated with 10 μl MTT (37°C; 3 h), according to the manufacturer’s instruction. The supernatant was removed, and the insoluble formazan crystals were dissolved in 100 μL of dissolving solution. The absorbance of each well was measured with a microplate reader at a wavelength of 570 nm.

### siRNA knockdown of TRPM4 expression

Small interfering RNA (siRNA) against the rat TRPM4 channel (siTRPM4; ON-TARGET plus SMARTpool Trpm4 siRNA; ID: L-101661-02-0005) and negative control siRNA (siNEG; siGENOME Non-Targeting siRNA Pool #1; ID: D-001206-13-05) were purchased from Thermo Scientific Dharmacon (Lafayette, CO, USA). H9c2 cells were seeded (20,000 cells / well) in a 96-well plate. The final concentration of siRNA was 100 nM diluted in OPTI-MEM medium. The cells were transfected with siRNA using DharmaFECT, according to the manufacturer’s protocol. After 24–48 h, the mRNA level of TRPM4 was determined by real-time PCR and Western blot.

### Quantification of TRPM4 mRNA using real-time PCR

Total RNA was extracted using the High Pure RNA Isolation Kit (Roche, Indianapolis, IN, USA), and reverse-transcribed into cDNA using the Verso cDNA Kit (Thermo Fisher Scientific, Vilnius, Lithuania). The cDNA was quantified by real-time PCR using SYBR Green reagents (Life Technologies, Warrington, UK), and primers specific for TRPM4 and GAPDH. The PCR primers specific for the TRPM4 channels were as previously described [12]: sense, 5′-AGTTGAGTTCCCCCTGGACT-3′; antisense, 5′-GAACTTGCCCCACATTAGGA-3′. The expected PCR product size was 148 base pairs. The PCR primers specific for GAPDH were: sense, 5′-ATGTTCCAGTATGACTCCACTCACG-3′, antisense, 5′-GAAGACACCAGTAGACTCCACGACA-3′. The expected PCR product size was 171 base pairs. The mRNA level of TRPM4 was determined by normalization against the expression level of GAPDH, and the relative expression was calculated according to the ΔCt method. All measurements were conducted in triplicates on five independent samples per treatment group. The data were expressed as fold increase relative to the control sample.

### Western blot

Cultured H9c2 cells were harvested, washed in cold PBS, and lysed with the Ambion KDalert Lysis Buffer (Life Technologies). Protein concentration was measured with the Protein Assay Rapid Kit (Wako, Osaka, Japan). Equal amounts of protein from each sample were separated by 8% SDS-PAGE (Bolt bis-tris Plus Gel, Life Technologies), and transferred onto a polyvinylidene difluoride (PVDF) membrane (Invitrogen, Tokyo, Japan). The non-specific sites were blocked by incubating the membrane (1 h, room temperature) in 3% (w/v) bovine serum albumin (BSA) added to Tris-buffered saline containing 0.005% (v/v) Tween-20 (TBS-T). Then, the membrane was incubated overnight (4°C) with TRPM4 antibody (1:250; ab63080, Abcam). The membrane was washed in TBS-T, and incubated with HRP-conjugated secondary antibody (1:50000) for 1 h. The membrane was washed with TBS-T, and specific proteins were visualized using an ECL Prime Detection System (GE Healthcare, Tokyo, Japan).

### Immunocytochemistry

Cells were fixed with 4% paraformaldehyde for 10 min at room temperature, permeabilized with cold methanol (−20°C; 10 min), blocked with 10% goat serum albumin for 1 h, and incubated with TRPM4 antibody (1:250; ab63080, Abcam) overnight at 4°C. Then, they were washed and incubated with Alexa Fluor 488-conjugated secondary antibody for 1 h at room temperature (1: 10000; ab150077, Abcam), washed once with PBS, and incubated with Hoechst 33342 (1:1000 dilution, Abcam) for 1 min to stain the nuclei. The cells stained only with the secondary antibody served as negative control. Fluorescence images were acquired with a confocal microscope (LSM 780, Zeiss, Germany). They were all blinded to the experimenter and acquired with identical parameters.

### Statistical Analysis

All data are expressed as the mean ± standard error of the mean (SEM) and were analyzed using Prism software (version 5.0, Graphpad Software, La Jolla, CA, USA). Comparisons between two groups were conducted using Student t-tests, whereas multiple comparisons were conducted by ANOVA followed by Bonferroni post-hoc test unless otherwise indicated. Statistical significance was established for *p* values <0.05.

## Results

### 9-Phe reduces myocardial infarction area in vivo

**Fig 2:**
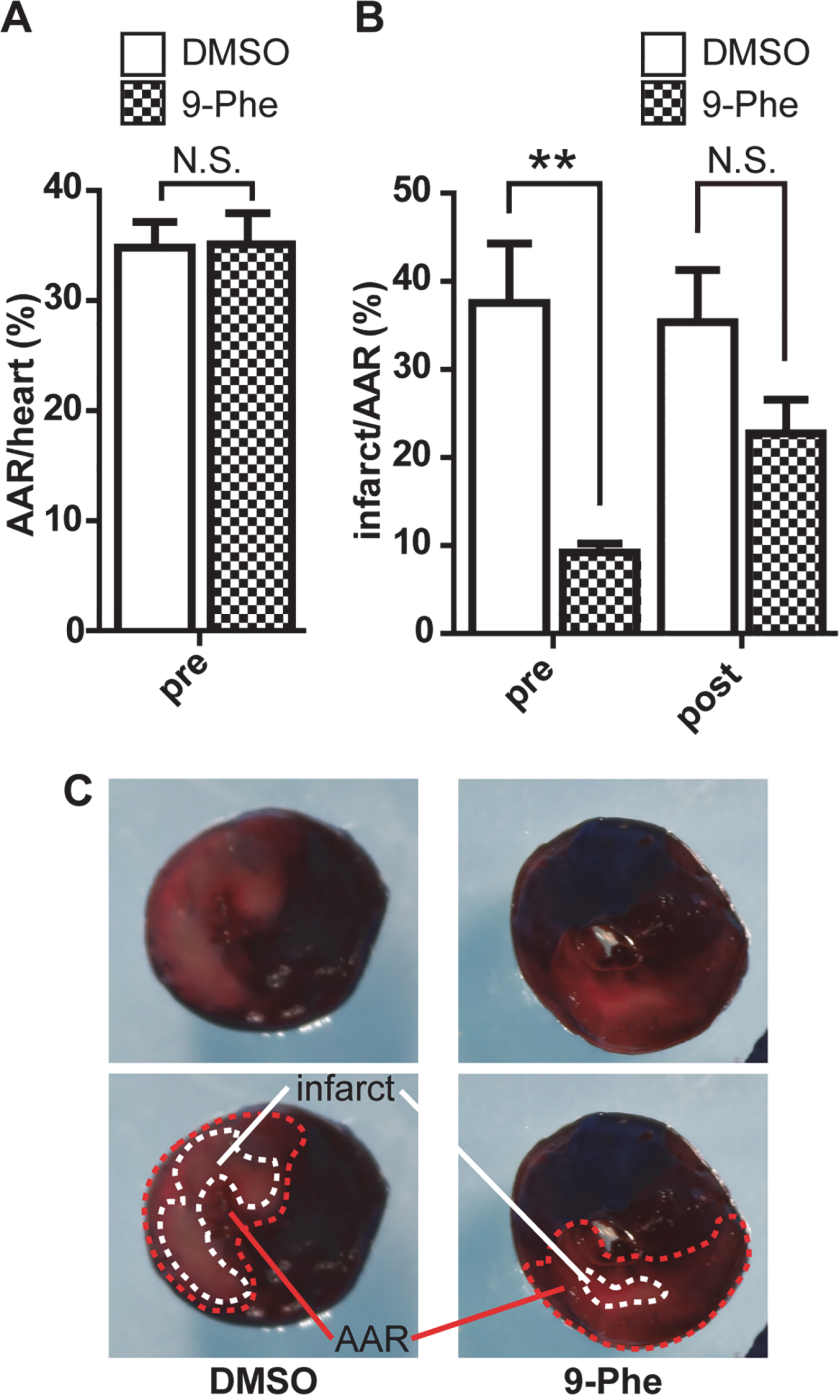
Impact of 9-Phe on the size of myocardial infarction. Rats received a bolus injection of DMSO (control) or 9-Phe before (preconditioning) or after (postconditioning) ischemia during an ischemia/reperfusion (I/R) protocol. **(A)** Impact on the percentage of area at risk (AAR) caused by I/R. **(B)** Impact on the percent infarct size over AAR. A fixed detection threshold for infarcted area was arbitrarily set, and used throughout the analysis. Only 9-Phe preconditioning significantly reduced the percent infarcted size, compared to DMSO (n = 5–6; *p* < 0.01). **(C)** Typical TTC-stained heart slices after preconditioning with DMSO or 9-Phe. The blue region indicates cardiac tissue that received normal blood flow, whereas the red region indicates ischemic tissue due to LAD occlusion. The light red region encircled by a dotted line indicates the infarcted tissue.

Animal studies were conducted to determine whether 9-Phe may protect the heart against I/R injury. Successful ischemic treatment by LAD occlusion was confirmed by Evans blue staining at the end of each experiment. 9-Phe preconditioning did not significantly affect the size of AAR compared with DMSO preconditioning (34.8 ± 2.6% and 35.1 ± 3.1%, respectively; Fig 2A). In contrast, 9-Phe preconditioning significantly reduced myocardial infarct size (% infarcted area over AAR) (Fig 2B and 2C). The infarcted region was 4-fold smaller in the 9-Phe group than in the DMSO group (9.2 ± 1.1% and 37.5 ± 7.6%, respectively; *p* < 0.01).

Next, we tested whether 9-Phe has a cardioprotective effect when applied just before the reperfusion procedure (postconditioning) (Fig 2B). The percent infarcted area in the 9-Phe group (22.8 ± 3.8%, n = 6) was nonsignificantly smaller than that in the DMSO group (35.4 ± 5.9%, n = 6).

Altogether, these data suggest that an injection of 9-Phe before (not after) myocardial ischemia could considerably suppress I/R-induced cardiac infarction.

### Expression of TRPM4 in the rat heart

**Fig 3:**
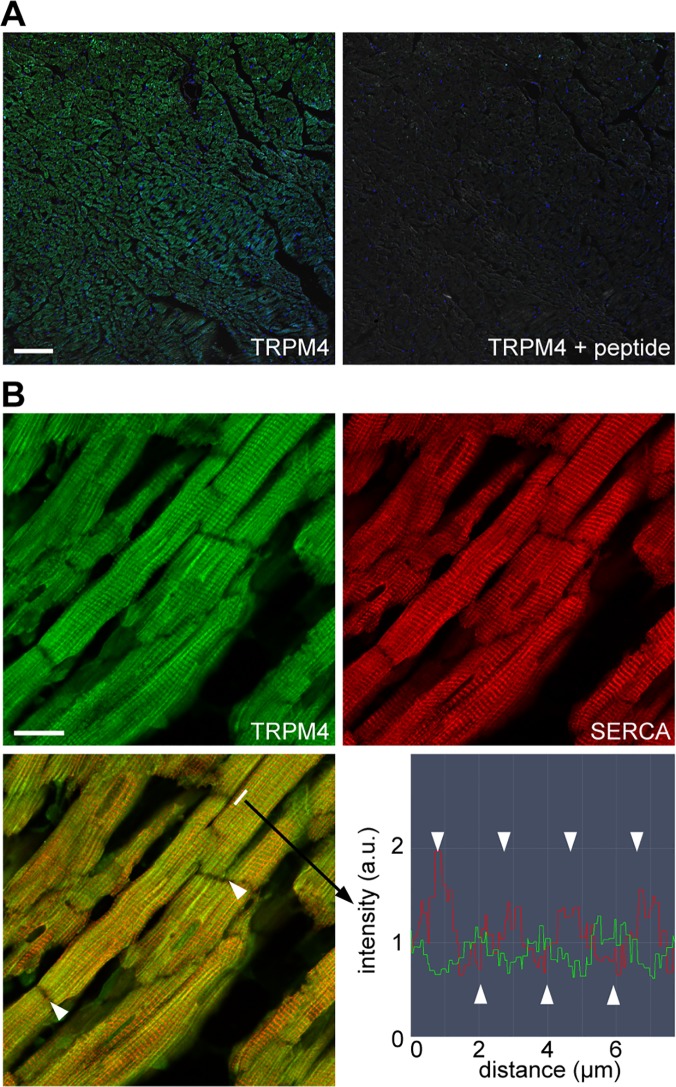
Immunofluorescence imaging of TRPM4 in the healthy rat heart. **(A)** Adult rat heart sections were labeled with anti-TRPM4 antibodies (green) and stained with Hoechst 33342 for the nuclei (blue). The green and blue fluorescence images were overlaid with the DIC image. Staining specificity was confirmed by the lack of signal when anti-TRPM4 antibodies were preincubated with the antigenic blocking peptide (ab65597). Scale bar: 100 μm. **(B)** Double immunofluorescence showing TRPM4 (green) and SERCA2 (red) expression in ventricular cardiomyocytes. Scale bar: 20 μm. The overlaid image (left bottom) reveals alternating positions for the TRPM4 and SERCA2 proteins on the longitudinal axis of cardiomyocytes. Arrowheads indicate the intercalated disc. Fluorescence intensity profile (right bottom) confirmed the alternating expression of TRPM4 and SERCA2.

The expression of TRPM4 in the rat heart was confirmed by immunohistochemistry and immunofluorescence assay. Positive TRPM4 staining was observed in the ventricle and atrium (S1 Fig). In the DAB staining, the signal was less intense in the kidney, liver, lung, and pancreas than in the ventricle and atrium. Similar results were obtained using a different anti-TRPM4 antibody. DAB staining was not observed in the sections that were not exposed to the primary antibody. In addition, we tested specificity of the primary antibody using the antigenic blocking peptide. The fluorescence significantly decreased in the presence of the antigenic blocking peptide (Fig 3A). Next, the localization of TRPM4 in the rat heart tissue was investigated with the use of double immunofluorescence assay (anti-TRPM4 and SERCA2). Fig 3B shows a striated pattern of alternating TRPM4-positive and SERCA2-positive bands in the longitudinal section. TRPM4 was not expressed in the intercalated discs.

### Protective effect of 9-Phe against H_2_O_2_ in H9c2 cardiomyocytes

**Fig 4:**
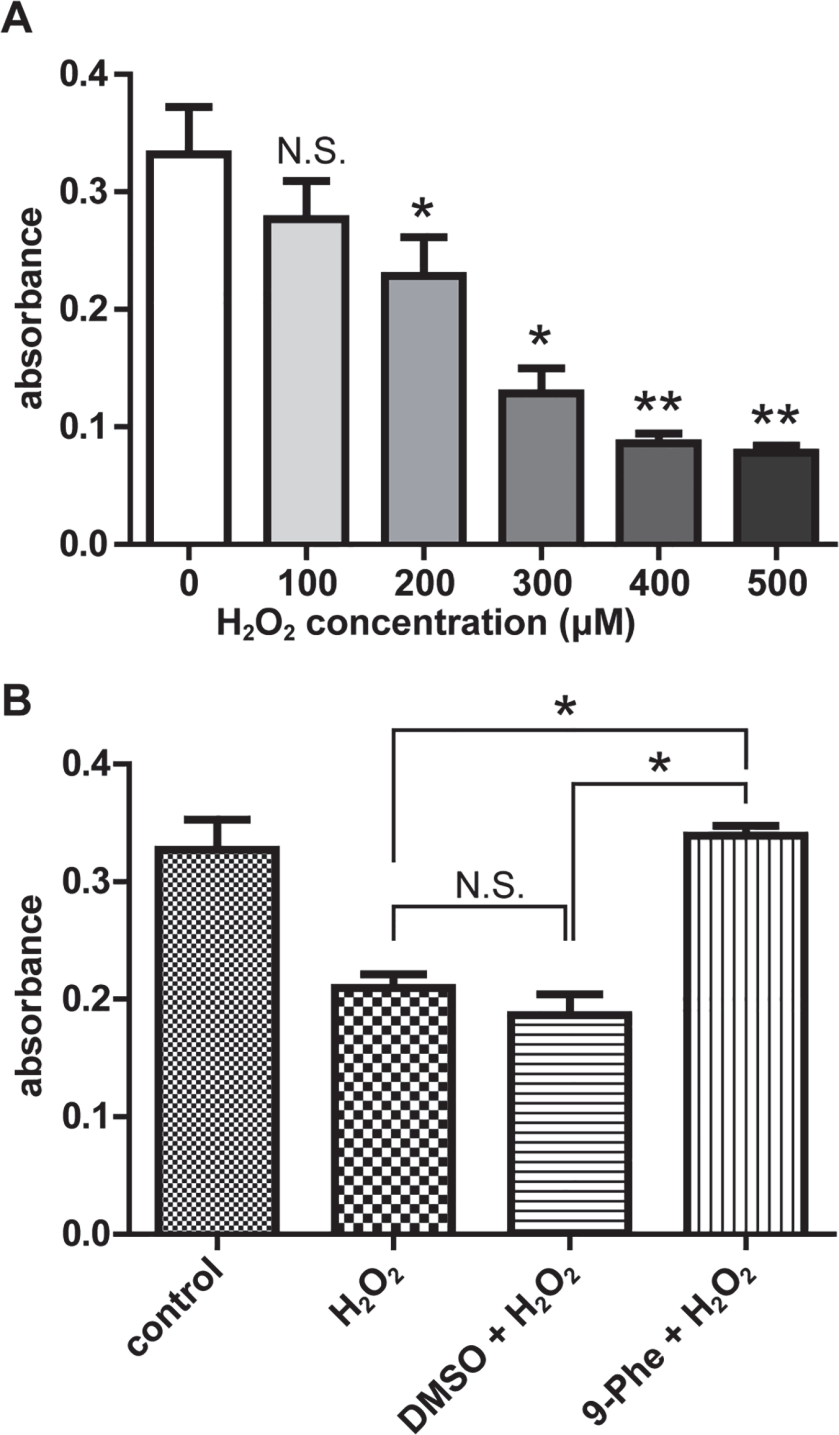
Protective effect of 9-Phe against H_2_O_2_-induced death in H9c2 cardiomyocytes. **(A)** Dose-dependent impact of H_2_O_2_ on cell viability. Cultures of H9c2 cells were exposed 4 h to DMEM medium containing 0, 100, 200, 300, 400, or 500 μM H_2_O_2_, then viability was measured by MTT assay. Asterisks indicate significantly lower viability (absorbance) than for the control (0 μM H_2_O_2_). n = 4 for each concentration. **(B)** Impact of 9-Phe on the H_2_O_2_ challenge. Cultures were incubated 4 h with 200 μM H_2_O_2_ in the presence of DMSO or 20 μM 9-Phe. The 9-Phe completely prevented the damage caused by H_2_O_2_. n = 5 for each condition. * *p* < 0.05, ** *p* < 0.01, N.S.: *p* > 0.05.

The protective effect of 9-Phe against ROS-induced injury was investigated by exposing H9c2 cardiomyocytes to H_2_O_2_. Fig 4A shows that a 4-h exposure to H_2_O_2_ (0–500 μM) caused a concentration-dependent decrease in cell viability. The minimum effective dose was 200 μM H_2_O_2_. Fig 4B shows that 200 μM H_2_O_2_ reduced the viability of H9c2 cells by nearly 40% in the H_2_O_2_ group (0.21 ± 0.01) and the DMSO + H_2_O_2_ group (0.19 ± 0.02) compared to the control group (0.33 + 0.02) (Fig 4B; *p* < 0.05). In contrast, pretreatment with 20 μM 9-Phe effectively protected the viability of the H9c2 cells, with a mean absorbance (0.34 ± 0.01) comparable to the control group. The dose-dependent protective effect of 9-Phe was tested using 1, 2, 5, and 10 μM of 9-Phe during 200 μM H_2_O_2_ challenges (S2 Fig). Cell viability was significantly improved only in the group treated with 20 μM 9-Phe (*p* < 0.05).

### Protective effect of 9-Phe against H/R-induced damage in H9c2 cardiomyocytes

**Fig 5:**
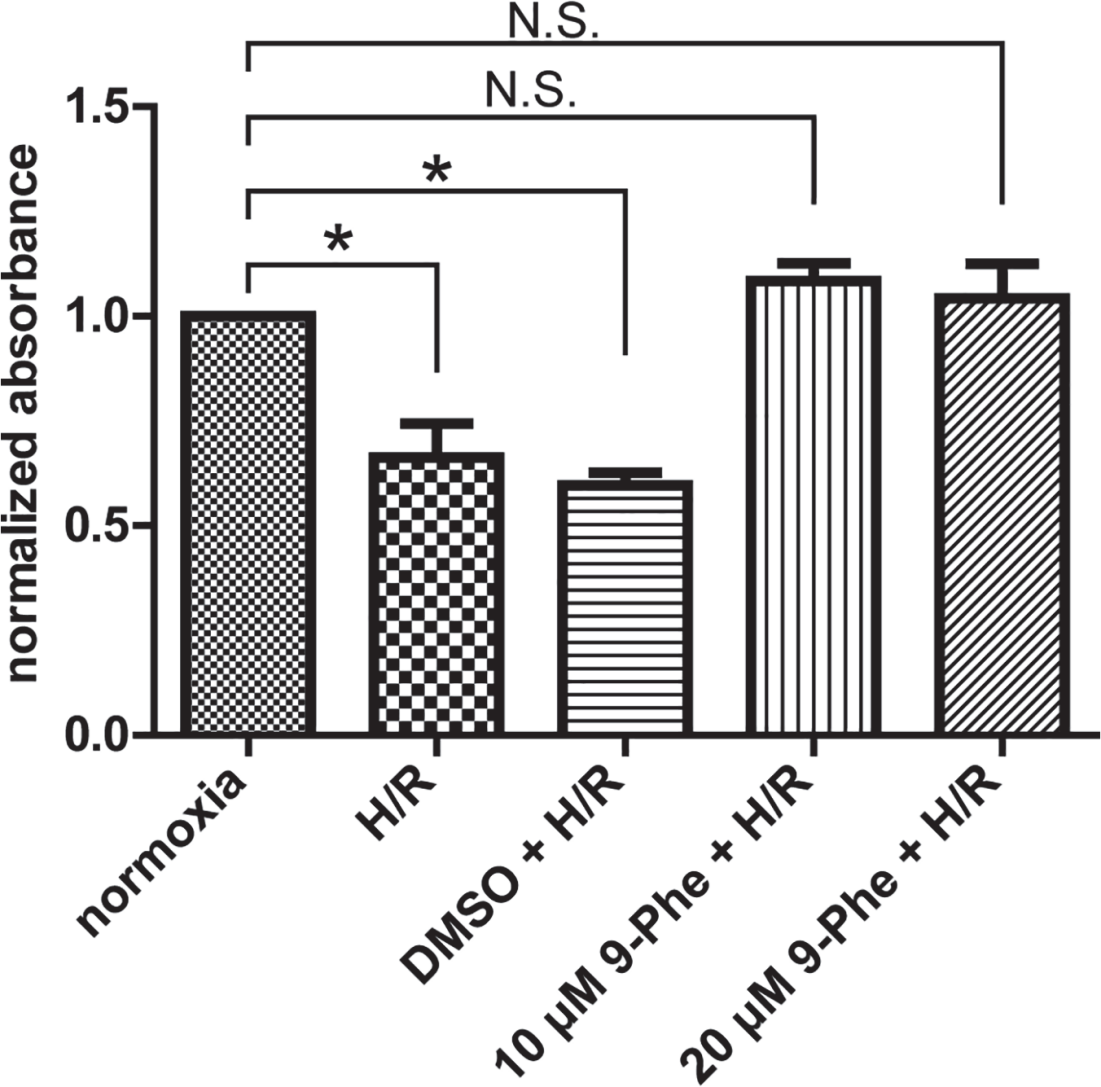
Protective effect of 9-Phe against hypoxia-reperfusion-induced death in H9c2 cardiomyocytes. Cultures of H9c2 cells, incubated with DMSO or 9-Phe (10 or 20 μM), were subjected to hypoxia-reperfusion (H/R; 4 h anoxia followed by 1 h reoxygenation). Viability was measured by the MTT assay, and absorbance was normalized to that of the normoxic condition. The presence of 9-Phe (≥10 μM) completely prevented the damage caused by H/R (n = 3 for each condition). Dunnett’s post hoc test was performed. * *p* < 0.05, N.S.: *p* > 0.05.

The cardioprotective effect of 9-Phe against H/R-induced injury was investigated in H9c2 cardiomyocytes exposed 4 h to anoxia followed by 1 h reoxygenation. Fig 5 shows that H/R significantly reduced the normalized absorbance of the H/R group (0.66 ± 0.10) and the DMSO + H/R group (0.60 ± 0.04) (one-way ANOVA with Dunnett’s post hoc test), compared to the normoxia group. In contrast, pretreatment with 10 μM and 20 μM of 9-Phe maintained normal cell viability (1.04 ± 0.10 and 1.08 ± 0.05, respectively). These data suggest that 9-Phe pretreatment can effectively protect cardiomyocytes against H/R-induced injury.

### Knockdown of TRPM4 prevents H_2_O_2_-induced cell death in H9c2 cardiomyocytes

**Fig 6:**
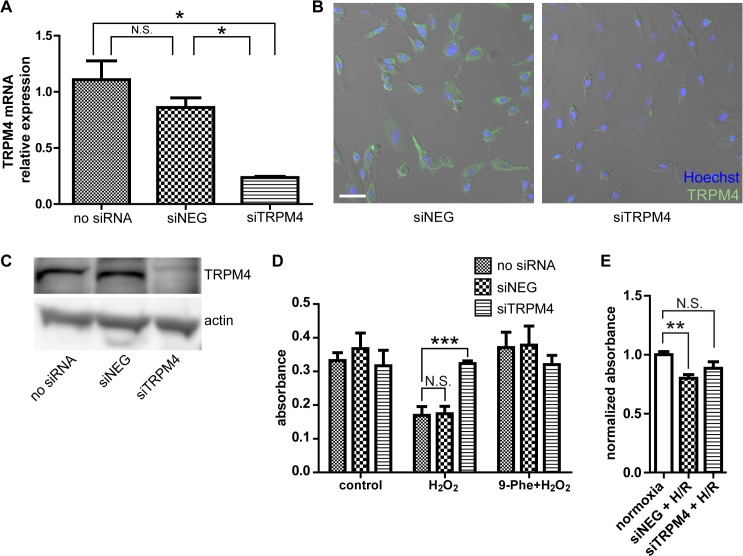
Knockdown of TRPM4 prevents cell death caused by H_2_O_2_ challenge in H9c2 cardiomyocytes. **(A)** Quantitative RT-PCR confirming the gene silencing of TRPM4. siNEG, cells transfected with control siRNA; siTRPM4, cells transfected with TRPM4-targeting siRNA. n = 5 for each group. **(B)** Confirmation of suppressed TRPM4 protein expression by immunocytochemistry 48 h after siRNA transfection. Green, anti-TRPM4, Blue, Hoechst 33342 dye (nuclei). The fluorescent images were overlaid with DIC images of the cultures. **(C)** Confirmation of suppressed TRPM4 protein expression by Western blot 24 h after siRNA transfection. **(D)** Impact of gene silencing on the loss of viability induced by 200 μM H_2_O_2_. Cell viability was measured by the MTT assay. n = 5 for each group. **(E)** Impact of TRPM4 knockdown on the hypoxia/reoxygenation (H/R) challenge. Cell viability was measured by the MTT assay. n = 6 for each group. Statistical analysis was performed using Dunnett’s test as post hoc. *: *p* < 0.05, **: *p* < 0.01, N.S.: *p* > 0.05.

The possible involvement of TRPM4 channel in the cardioprotective effect of 9-Phe against oxidative stress was investigated by gene silencing of TRPM4 in H9c2 cells. The efficiency of the siRNA transfection was tested at the mRNA and protein levels. Fig 6A shows that siRNA targeting TRPM4 reduced the mRNA level of TRPM4 to 20% in the siTRPM4 group (0.24 ± 0.02), compared to the control cells (no siRNA; 1.10 ± 0.29, *p* < 0.05). The fact the mRNA level was only reduced to 90% of controls in the siNEG groups (0.86 ± 0.15) demonstrates the selectivity of the silencing protocol. The impact of gene silencing on TRPM4 protein expression was determined by immunocytochemistry (Fig 6B). Whereas the siNEG transfected cultures showed distinct TRPM4-positive cells, signals were hardly detected in cells transfected with siTRPM4. Similar results were obtained by Western blot experiments (Fig 6C). Collectively, these data show that gene silencing significantly reduced the expression of TRPM4 channels.

The transfection of siNEG or siTRPM4 did not significantly alter the viability of H9c2 cells under control conditions (Fig 6D). Exposure of non-transfected cells to 200 μM H_2_O_2_ reduced cell viability by 50% (no siRNA, absorbance: 0.17 ± 0.03). The siNEG transfection did not protect the cells against H_2_O_2_. In contrast, H_2_O_2_ did not reduce the viability of TRPM4 knocked down cells (siTRPM4, absorbance: 0.32 ± 0.01). When the cells were pretreated with 20 μM of 9-Phe, H_2_O_2_ did not reduce cell viability, regardless of the siRNA transfection. Altogether, these data suggest that ROS-mediated injury in cardiomyocytes is mediated by TRPM4 channels and that the protective effects of 9-Phe are mediated by their inhibition. Effect of TRMP4 knockdown on H/R was also studied (Fig 6E). Compared to the normalized absorbance of 1.00 ± 0.06 in the normoxia group, normalized absorbance values of the cells transfected with siNEG and siTRPM4, in the H/R condition, were 0.80 ± 0.08 (*p* < 0.01) and 0.88 ± 0.14 (not significant), respectively (one-way ANOVA with Dunnett’s post-hoc test).

## Discussion

The present study investigated the impact of 9-Phe on myocardial I/R injury in rats and the cellular mechanism of cardioprotection in cardiomyocytes. We found that pretreatment with 9-Phe considerably reduced the myocardial infarct size *in vivo*. Immunohistochemistry showed that TRPM4 channels are expressed in ventricular cardiomyocytes, where I/R causes infarction. Furthermore, TRPM4 channels are critically involved in the cardioprotective effects of 9-Phe against oxidative stress caused by H_2_O_2_ in H9c2 cardiomyocytes.

We previously reported that 9-Phe exerts a cardioprotective effect against I/R injury in isolated rat hearts [8]. In the present study, the cardioprotective effect of 9-Phe was demonstrated *in vivo* by preconditioning rats with the compound before I/R injury, as evidenced by a significant reduction in the infarct area. In the clinical setting, it would be more beneficial that treatment after I/R injury reduces risk of myocardial infarction. Although we tested the possibility that postconditioning is effective for cardioprotection, difference in sizes of infarcted areas between DMSO treated control group and 9-Phe treated group was not statistically significant (Fig 2B). As the cellular mechanisms regarding ischemic pre- and post-conditioning are still under debate [13], the different effects of 9-Phe on pre- and post-conditioning should be clarified in the future research.

The cardioprotective effects of drugs against myocardial I/R injury are commonly tested using H_2_O_2_ exposures and H/R injury models *in vitro* [14, 15]. The H9c2 cardiomyocyte cell line exhibits morphological characteristics typical of immature embryonic cardiomyocytes, but they express the electrical and hormonal signaling pathways of adult cardiac cells [16]. Accordingly, these models were both used in the present study to study the cardioprotective effect of 9-Phe in H9c2 cardiomyocytes. We demonstrate that the addition of 20 μM of 9-Phe immediately before a 4-h exposure to 200 μM H_2_O_2_ or H/R completely prevented the loss of cell viability. These results support the protective effect of 9-Phe against ROS-induced death in H9c2 cardiomyocytes. However, 20 μM 9-Phe did not exhibit any protective effect against H/R in dissociated adult cardiomyocytes, and even decreased their viability. As this concentration of 9-Phe was found protective against I/R injury in the heart *ex vivo* [8], a direct toxicity of 9-Phe on intact heart is unlikely. On the other hand, this observation may eventually lead to a better understanding of the protective mechanism of this compound and the role of TRPM4 channels.

The cardioprotective mechanism of 9-Phe was investigated based on the known property of this compound as a selective inhibitor of TRPM4 channels [9, 10]. Several organs and cells express TRPM4 channels, including the kidneys, liver, pancreas, skeletal muscles [17], as well as non-excitable cells like leukocytes [18]. In the present study, immunolocalization confirmed the expression of TRPM4 proteins in ventricular cardiomyocytes. Furthermore, we showed that the TRPM4 channels are expressed alternately with the SERCA2 pump. Yoo et al. reported that TRPM4b is expressed not only in the plasma membrane but also in the endoplasmic reticulum in the transient expression system using COS-7 cells [19]. This study suggests that TRPM4 may be expressed in the sarcoplasmic reticulum of cardiomyocytes. We provide evidence that TRPM4 channels are not expressed in the intercalated discs of cardiomyocytes, where TRPC3 channels are highly expressed [20]. In addition, we showed that the protein expression of TRPM4 was higher in cardiac tissue than in the kidneys or liver. These findings are in agreement with the previous mRNA expression profiles reported for these organs [17, 21]. These data confirm that the target of 9-Phe is highly expressed in cardiomyocytes, thus justifying the use of the H9c2 cell line to investigate the mechanism of action of this compound.

The present study provides evidence that the cardioprotective effect of 9-Phe against oxidative stress involves an inhibition of TRPM4 channels. First, TRPM4 knockdown efficiently prevented H_2_O_2_-induced cell death in H9c2 cardiomyocytes. Second, in the absence of TRPM4 channels, 9-Phe had no effect on the H_2_O_2_-mediated reduction of cell viability. TRPM4 is a Ca^2+^-activated nonselective cation channel [17] recently found to be regulated by ROS [22, 23, 24]. In TRPM4-overexpressed HEK293 cells, H_2_O_2_ prevented TRPM4 desensitization [20]. The resulting continuous inward Na^+^ current caused cell swelling and death. In addition, TRPM4 was reported to play a vital role in LPS-induced endothelial cell death, by a mechanism involving LPS-induced excess ROS production [23]. Collectively, these findings suggest that the remarkable cardioprotective effect of 9-Phe against oxidative stress injury is mediated by TRPM4 inhibition.

TRPM4 channels are also involved in calcium homeostasis [25]. Their opening facilitates membrane depolarization and activates voltage-gated calcium channels, which may lead to calcium overload in the ventricular cardiomyocytes. In the present study, blockade of TRPM4 channels by 9-Phe prevented ischemia-reperfusion injury *in vivo*. It is tempting to speculate that the inhibition of TRPM4 activity may prevent calcium overload during ischemia-reperfusion injury. However, further study is needed to test this hypothesis.

In conclusion, this study suggests that 9-Phe protects cardiomyocytes against I/R injury *in vivo* and *in vitro* by a mechanism involving the inhibition of TRPM4 channels. Thus, the injection of TRPM4 blocker before myocardial reperfusion may constitute a new strategy to mitigate I/R injury in patients with ischemic heart disease.

## Supporting Information

S1 Fig Immunohistochemistry of TRPM4 expression in various organs. Organ sections were stained with anti-TRPM4 antibodies, and the brown areas are positive for TRPM4 staining. **(A)** TRPM4 expression in the rat ventricle, atrium, kidney, and liver. Ab (−): without primary antibody, Ab (+): with primary antibody. Scale for 5× magnification: 200 μm, Scale for 20× magnification: 50 μm. **(B)** The known tissue distribution of TRPM4 in respiratory epithelia and pancreatic islets was confirmed. Scale: 100 μm.
(TIF)

S2 Fig Dose-dependent protective effect of 9-Phe against H_2_O_2_-induced death in H9c2 cardiomyocytes. Cultures were exposed 4 h to 200 μM H_2_O_2_ in the presence of 9-Phe (0, 1, 2, 5, 10, or 20 μM). Then, cellular viability was measured by MTT assay. Asterisk indicates significant difference (*p* < 0.001) from the absorbance at 0 μM of 9-Phe. n = 8 for 0 μM and 20 μM of 9-Phe; n = 3 for 1, 2, 5, and 10 μM 9-Phe. Dunnett’s multiple post hoc test was used.
(TIF)

S3 Fig Lack of protective effect of 9-Phe against hypoxia-reperfusion-induced cell death in dissociated adult rat cardiomyocytes. Dissociated adult rat cardiomyocytes were subjected to normoxia or hypoxia-reperfusion (H/R; 4 h anoxia followed by 1 h reoxygenation) in the presence of DMSO or 20 μM 9-Phe (n = 3 for each group). Viability was measured by the MTT assay.
(TIF)

S1 Text (DOCX)
