# Supplementary figures and images for: Transient Receptor Potential Melastatin-4 Is Involved in Hypoxia-Reoxygenation Injury in the Cardiomyocytes
Source: PLoS One. 2015 Apr 2;10(4):e0121703. doi: 10.1371/journal.pone.0121703 (PMC4383534; doi:10.1371/journal.pone.0121703)

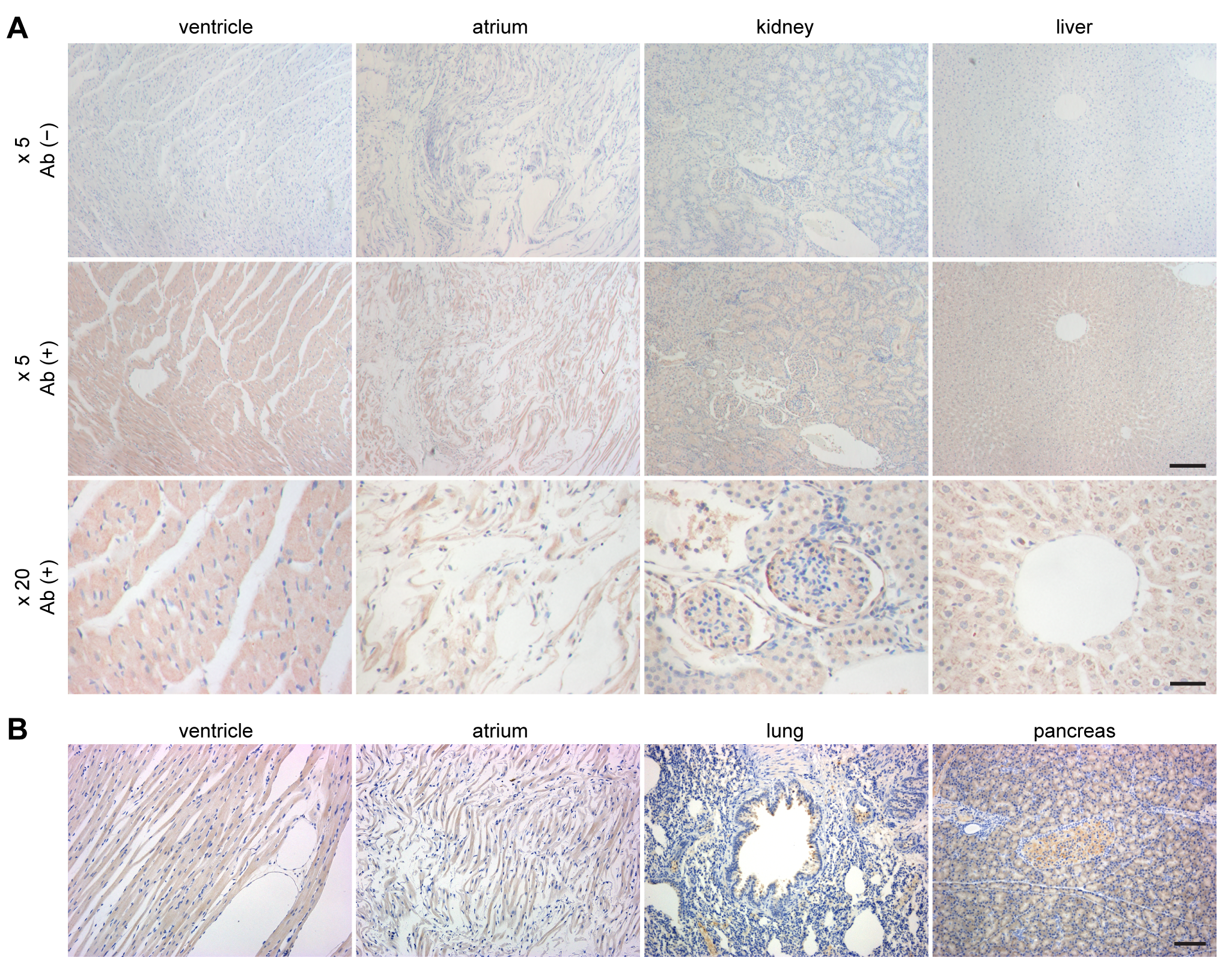

Supplement: S1 Fig — Organ sections were stained with anti-TRPM4 antibodies, and the brown areas are positive for TRPM4 staining. (A) TRPM4 expression in the rat ventricle, atrium, kidney, and liver. Ab (−): without primary antibody, Ab (+): with primary antibody. Scale for 5× magnification: 200 μm, Scale for 20× magnification: 50 μm. (B) The known tissue distribution of TRPM4 in respiratory epithelia and pancreatic islets was confirmed. Scale: 100 μm. (TIF) [file pone.0121703.s001.tif]

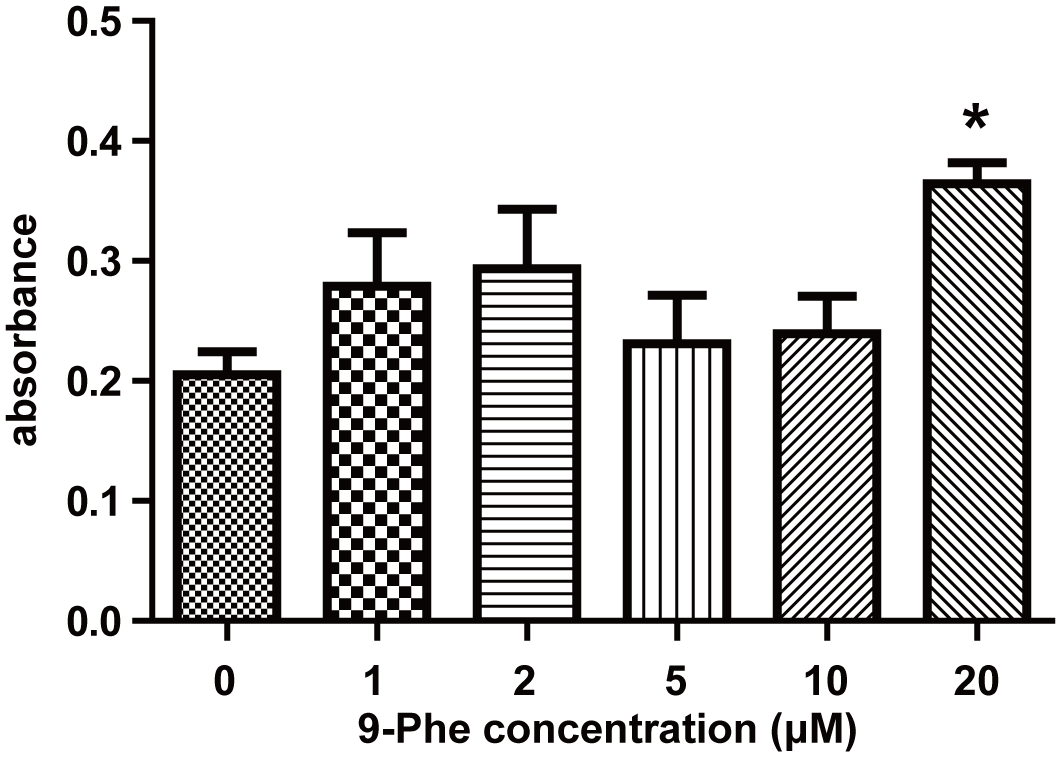

Supplement: S2 Fig — Cultures were exposed 4 h to 200 μM H2O2 in the presence of 9-Phe (0, 1, 2, 5, 10, or 20 μM). Then, cellular viability was measured by MTT assay. Asterisk indicates significant difference (p < 0.001) from the absorbance at 0 μM of 9-Phe. n = 8 for 0 μM and 20 μM of 9-Phe; n = 3 for 1, 2, 5, and 10 μM 9-Phe. Dunnett’s multiple post hoc test was used. (TIF) [file pone.0121703.s002.tif]

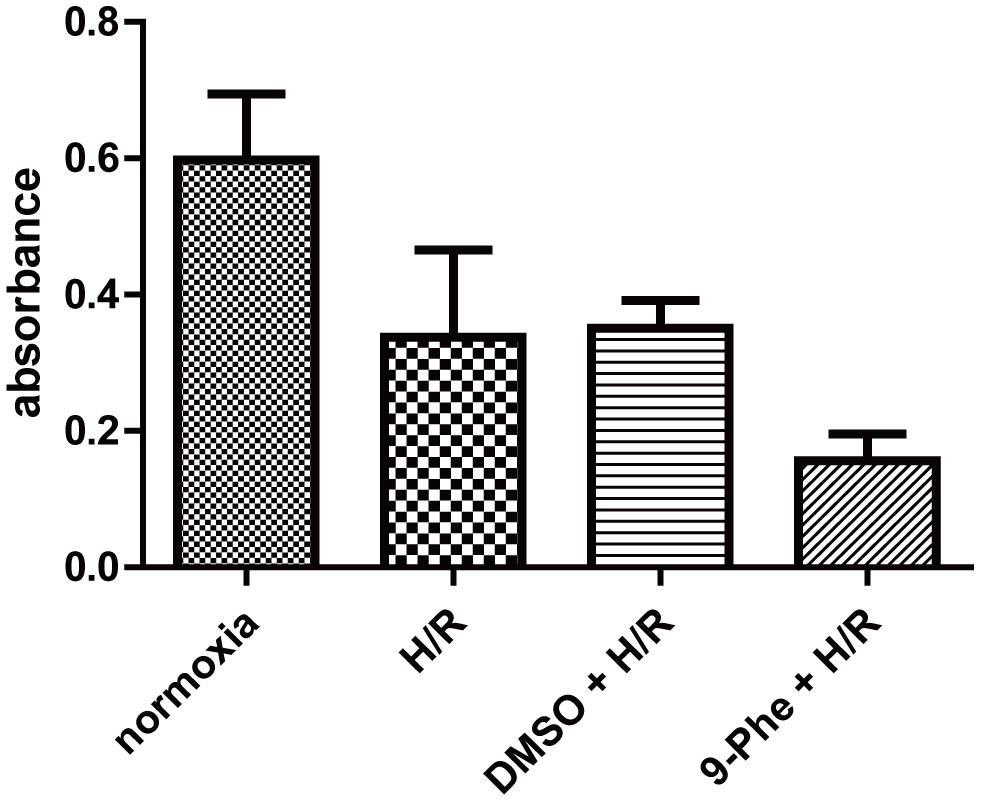

Supplement: S3 Fig — Dissociated adult rat cardiomyocytes were subjected to normoxia or hypoxia-reperfusion (H/R; 4 h anoxia followed by 1 h reoxygenation) in the presence of DMSO or 20 μM 9-Phe (n = 3 for each group). Viability was measured by the MTT assay. (TIF) [file pone.0121703.s003.tif]
