# Supplementary material for: Transient Receptor Potential Melastatin-4 Is Involved in Hypoxia-Reoxygenation Injury in the Cardiomyocytes
Source: PLoS One. 2015 Apr 2;10(4):e0121703. doi: 10.1371/journal.pone.0121703 (PMC4383534; doi:10.1371/journal.pone.0121703)
Supplement: S1 Text — (DOCX) [file pone.0121703.s004.docx]

**Supplementary method**

Preparation of adult rat cardiomyocytes

Ventricular myocytes were isolated enzymatically from hearts excised from 12–15 weeks old male Sprague Dawley rats. The procedures are described elsewhere [1], except that the perfusion speed was set to 6.5 mL/min. The cells were re-suspended in Dulbecco’s modified Eagle’s medium (D-MEM) with 10% FBS, and stored in a CO_2_ incubator before use.

*Exposure to hypoxia/reoxygenation*

The cardiomyocytes were incubated in serum-free medium under hypoxic conditions (95% N_2_; 5% CO_2_) for 4 h at 37°C. Then, the cells were cultured in DMEM supplemented with 10% FBS under normoxic conditions for 1 h. Cellular viability was measured using MTT assay.

**Supplementary reference**

1. G. Iribe, K. Kaihara, H. Ito, K. Naruse. (2013) Effect of azelnidipine and amlodipine on single cell mechanics in mouse cardiomyocytes. Eur. J. Pharmacol., 715, pp. 142–146.
